# Investigations on the Electrochemical Atomic Layer Growth of Bi_2_Se_3_ and the Surface Limited Deposition of Bismuth at the Silver Electrode

**DOI:** 10.3390/ma11081426

**Published:** 2018-08-14

**Authors:** Walter Giurlani, Andrea Giaccherini, Nicola Calisi, Giovanni Zangari, Emanuele Salvietti, Maurizio Passaponti, Stefano Caporali, Massimo Innocenti

**Affiliations:** 1 Dipartimento di Chimica, Università degli Studi di Firenze, via della Lastruccia 3, 50019 Sesto Fiorentino, Italy; emanuele.salvietti@unifi.it (E.S.); maurizio.passaponti@unifi.it (M.P.); 2 Consorzio Interuniversitario Nazionale per la Scienza e Tecnologia dei Materiali, via Giusti 9, 50121 Florence, Italy; andrea.giaccherini@unifi.it (A.G.); nicola.calisi@unifi.it (N.C.); stefano.caporali@unifi.it (S.C.); 3 Dipartimento di Scienze della Terra, Università degli Studi di Firenze, via La Pira 4, 50121 Firenze, Italy; 4 Dipartimento di Ingegneria Industriale, Università degli Studi di Firenze, via S. Marta 3, 50139 Florence, Italy; 5 Department of Materials Science and Engineering, University of Virginia, Charlottesville, VA 22904, USA; gz3e@virginia.edu; 6 Istituto dei Sistemi Complessi, Consiglio Nazionale delle Ricerche, via Madonna del Piano 10, 50019 Sesto Fiorentino, Italy; 7 Istituto di Geoscienze e Georisirse, Consiglio Nazionale delle Ricerche, via La Pira 4, 50121 Florence, Italy

**Keywords:** bismuth, bismuth selenide, topological insulator, E-ALD, UPD

## Abstract

The Electrochemical Atomic Layer Deposition (E-ALD) technique is used for the deposition of ultrathin films of bismuth (Bi) compounds. Exploiting the E-ALD, it was possible to obtain highly controlled nanostructured depositions as needed, for the application of these materials for novel electronics (topological insulators), thermoelectrics and opto-electronics applications. Electrochemical studies have been conducted to determine the Underpotential Deposition (UPD) of Bi on selenium (Se) to obtain the Bi_2_Se_3_ compound on the Ag (111) electrode. Verifying the composition with X-ray Photoelectron Spectroscopy (XPS) showed that, after the first monolayer, the deposition of Se stopped. Thicker deposits were synthesized exploiting a time-controlled deposition of massive Se. We then investigated the optimal conditions to deposit a single monolayer of metallic Bi directly on the Ag.

## 1. Introduction

Bismuth (Bi) and antimony chalcogenides, where the chalcogenide is selenium (Se) or tellurium (Te), exhibit excellent thermoelectric properties, achieving thermoelectric figure of merit (ZT) values of about 1 [1]. These materials commonly crystallize in a rhombohedral structure and exhibit semiconducting behavior (band gap ~0.3 eV). Beyond thermoelectric behavior, these materials show an insulating response in the bulk material while exhibiting metallic conductivity at grain boundaries or surfaces; surface states in particular, are protected via time reversal symmetry, such that electron scattering does not occur [2]. Recently, it has also been shown that the electrochemical growth of Bi_2_Se_3_ at low temperatures results in the formation of an orthorhombic structure with a higher band gap of about 1.1 eV [3,4]. Of interest, in the context of light absorbers and solar cells, the same behavior was also obtained by growing Bi_2_Se_3_ via the Successive Ionic Layer Adsorption and Reaction (SILAR) method [5]. 

The layered structure of the rhombohedral crystal, with Van der Waals interactions between the layers, encourages synthetic opportunities for the direct growth of layered structures or two-dimensional materials. In this study we evaluate the possibility to exploit the Electrochemical Atomic Layer Deposition (E-ALD) method [6,7] to synthesize these kind of materials. E-ALD is a widely known method capable of finely controlling the deposition process, allowing the growth at room temperature and pressure of highly ordered crystalline structures, starting from the first atomic layers, without the need for any further treatment (e.g., thermal annealing). These characteristics could overcome the problems of internal stresses, low crystallinity and coverage, evidenced in previous studies [6,7].

E-ALD exploits Surface-Limited Reactions (SLRs), such as Underpotential Deposition (UPD), thus achieving the layer-by-layer growth of various compound semiconductors [8,9,10,11,12,13,14,15,16,17]. The Nernst equation predicts the redox potential necessary to reduce metal ion A onto a substrate of the same element A. In the case where element A is deposited on a different substrate B, where the bonding strength of A–B is stronger than A–A, the potential needed for the deposition is more positive than the value predicted by the Nernst equation, thus resulting in a UPD process. This phenomenon enables the formation of sub-monolayers or a full atomic monolayer of A onto B. In fact, a second layer cannot be deposited at the same underpotential since the surface layer is now A, featuring a deposition potential dictated by the Nernst equation that is more negative. This would lead to a Surface-Limited Reaction (SLR) that spontaneously generates a single atomic layer. This behavior could be exploited to form a metal alloy, bi or multilayered, if A and B are both metal. If A and B are instead a metal and a nonmetal respectively, the UPD process may form a semiconductor compound. The E-ALD process takes places when the deposition is performed alternating one element over the other to artificially synthesize a new material.

In a previous study, Zhang obtained relevant results in an acidic medium using a polycrystalline Pt electrode [18]; unfortunately, in the reported conditions the simultaneous bulk deposition of the two elements was possible, providing some unwanted bulk 3D structure. The final compound, in the orthorhombic phase, showed a bandgap of 0.3 eV, typical of the rhombohedral phase and much lower than the expected value of 1.1 eV expected for the orthorhombic phase. In this work we discuss and describe synthetic efforts towards the preparation of Bi_2_Se_3_ both as a single monoatomic layer and thin film by the alternate deposition of Bi and Se monoatomic layers at the silver single crystal electrode in an alkaline buffer.

## 2. Materials and Methods

### 2.1. E-ALD Growth

All the solutions involved in this study were prepared with MilliQ water (18 MΩ, Merck Millipore, Burlington, MA, USA). The stock solution of the precursor for Bi and Se was prepared in a buffer solution. The latter was obtained by mixing NH_3_ 28% (Merck Millipore, Burlington, MA, USA) and HClO_4_ 65% (Carlo Erba, Cornaredo, Italy) solutions (pH = 9.13). The Na_2_SeO_3_ (Alfa Aesar, Haverhill, MA, USA) stock solution (1 mM) was used as a source for Se. Similarly, the precursor for Bi was a 0.5 mM stock solution of Bi(NO_3_)_3_ (Alfa Aesar, Haverhill, MA, USA) and 1 mM ethylenediaminetetraacetic acid disodium salt dihydrate (EDTANa_2_) (Alfa Aesar, Haverhill, MA, USA). Moreover, a 1 mM solution of EDTANa_2_ was used for stripping studies of Bi. In the case of Se stripping studies we used 0.1 M NaOH (Merck Millipore, Burlington, MA, USA) solution.

The stock solutions were stored in separated Pyrex jars filled with nitrogen and connected to a flow-cell through a circuit regulated by electro-valves. In order to circulate the desired solutions in the cell we used our homemade automated deposition system [19], connected to a computer, that controls the dedicated electro-valves. The pressure in the jar is adjusted to a solution flow rate of 1.5 mL/s. The electrolytic cell is a Kel-F cylinder with a capacity of 1.88 mL. The working electrode is a silver (Ag) (111) monocrystalline electrode (0.785 cm^2^) prepared and polished according to literature procedures [20]. The surface of this electrode is smooth; the roughness was investigated in previous studies from our group, and a roughness, measured as the root mean squared (RMS), less than 3 nm was measured [21]; polycrystalline gold was used as a counter electrode and Ag/AgCl sat. KCl as a reference electrode.

### 2.2. Spectroscopic Characterization

X-ray Photoelectron Spectroscopy (XPS) was used to evaluate the composition of the deposit obtained under different electrochemical conditions. The instrument makes use of a non-monochromatic X-ray source (VSW Scientific Instrument Limited model TA10, Manchester, UK), Mg Kα radiation (1253.6 eV), operating base at 120 W (12 kV and 10 mA) and a hemispherical analyzer (VSW Scientific Instrument Limited model HA100, Manchester, UK). The analyzer was equipped with a 16-channel detector and a dedicated differential pumping system that allows work during the acquisition of pressure in the main chamber up to the 10^−8^ mbar range. The pass energy was set to 22 eV. The measured spectra were analyzed using CasaXPS software (version 2.3.19, Casa Software Ltd, Teignmouth, UK). The inelastic background was subtracted using Shirley’s method [22] and a mixed Gaussian and Lorentzian contributions were used for each component. Calibration of the spectra was obtained by shifting to 284.8 eV, the lowest component relative to the 1s transition of carbon for adventitious carbon [23].

## 3. Results

**Figure 1:**
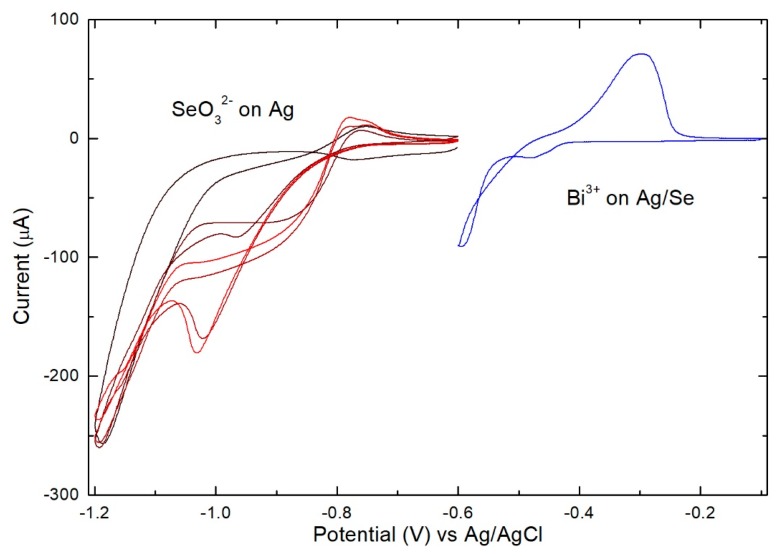
On the left: 4 consecutive CV of selenite (1st black–4th red) between −0.6 and −1.2 V, scan rate 50 mV/s. On the right: CV of the Bi solution on Ag/Se_ad_ between −0.1 and −0.55 V at 10 mV/s.

In order to alternately deposit monolayers of Se and Bi it is necessary to determine the condition for the SLR deposition of Bi on Ag (111) and on Ag (111)/Se_ad_ (the adlayer of Se). The deposition of Se monolayers has been reported in the literature [24] and consists of biasing the potential at −0.90 V for one minute in the presence of the selenite solution and then carrying out an ammonia buffer wash while maintaining the same potential for another minute. After covering the surface of the single crystal Ag electrode with a Se monolayer, a cyclic voltammetry of the Bi(NO_3_)_3_ solution (Figure 1) is carried out. The presence of a reductive peak that anticipates the massive deposition of the metal suggests the occurrence of a SLR.

**Figure 2:**
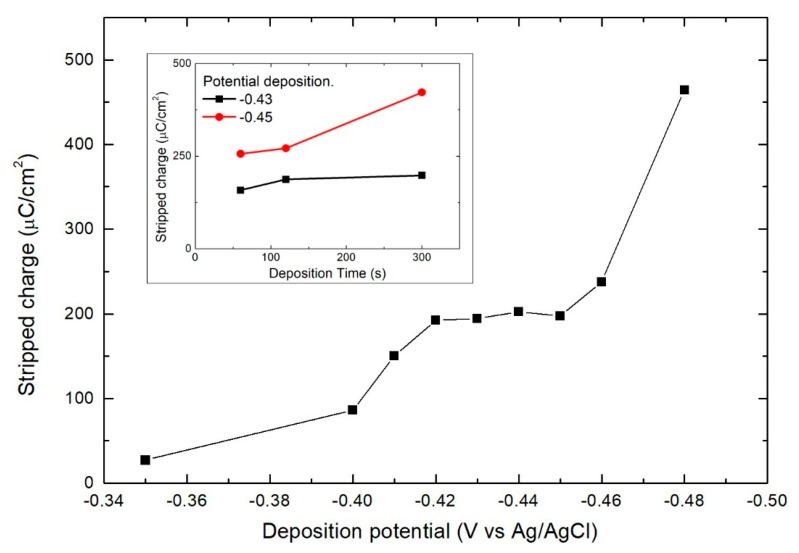
Growth of the amount of Bi deposited in one minute on Ag/Se_ad_ as a function of the deposition potential. In the inset, amount of deposited Bi with the potential fixed at −0.43 V (black) and −0.45 V (red) as a function of the deposition time.

Successively, we proceeded with a systematic study to find the best conditions for the SLR deposition of the metal. Bi depositions were carried out for one minute by varying the potential between −0.35 V and −0.48 V and then the deposit was anodically stripped in an EDTANa_2_ solution to evaluate the actual deposited amount (Figure 2). With increasingly negative stripping potentials, the charge increases, since the deposition time is not sufficient to deposit an entire monolayer of the metal, down to −0.42 V. However, at a potential more negative than −0.45 V, the charge grows very quickly indicating the onset of massive deposition. The optimal conditions for SLR deposition are most likely within the plateau between −0.42 V and −0.45 V. For further confirmation we repeated the depositions at −0.43 V and −0.45 V by varying the deposition time (inset in Figure 2). While at −0.43 V, the charge remained essentially constant, increasing the deposition time. At −0.45 V, the deposit tended to grow, indicating a slow deposition of massive Bi. For this reason, −0.43 V represents the optimal potential for the SLR deposition of Bi on Se.

Once the SLR conditions have been verified for both Se and Bi, several cycles were performed to obtain a deposit thicker than a single monolayer, following the procedure below:

- Se solution at −0.90 V for 60 s (growth of Se)
- Buffer solution at −0.90 V for 60 s (excess Se removal and rinse)
- Bi solution at −0.43 V for 60 s (growth of Bi)
- Washing with buffer solution (rinse)

**Figure 3:**
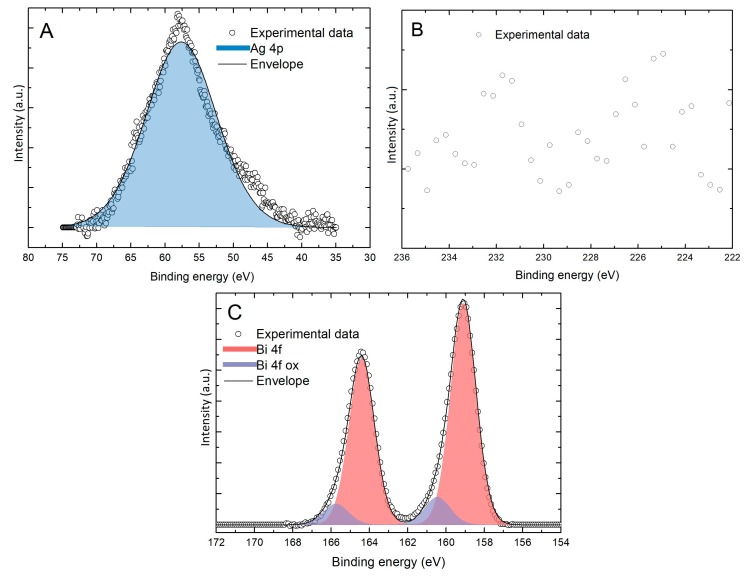
XPS peak of (**A**) 3d transition region of Se (not present) and Ag 4p; (**B**) 3s transition region of Se (not present) and (**C**) 4f transition region of Bi in the sample prepared performing 20 deposition cycles.

A sample was prepared by performing 20 deposition cycles and then analyzed by XPS (Figure 3). The peak related to the 4p transition of the silver substrate overlaps the Se 3d peak impairing the use of the latter to determine the amount of selenium in the sample. For this reason, in this work, we acquired both the regions of the 3d transition and 3s transition of Se. The first feature was used to evaluate the chemical shift for the evaluation of the chemical state of this element and the second was used for its quantification. The quantification and evaluation of the chemical shift of Bi was obtained using the 4f transition. In Figure 3 the absence of Se was confirmed from XPS analysis, in the region from 80 to 30 eV (Se 3d transition region). Only the Ag 4p transition was observed. In order to confirm this datum any signal was observed in the region from 236 to 222 eV (Se 3s transition region). The 4f transition of Bi was observed at 159.1 eV, and this position is compatible with Bi oxide (Bi_2_O_3_) [25,26]. These results show that the desired compound had not been formed and that the deposit had not grown as desired. This is not new, there are other literature examples (e.g. InAs [27] and SnS_x_ [28]) in which, even if they show a SLR, after a few cycles the growth spontaneously stops, impairing the formation of thin films. In such cases, this experimental evidence has been related to the intrinsic semiconductor nature, resulting in a material with limited conductivity (increased ohmic drop) and therefore hindering electron transfer, or due to displacement by the applied deposition potential.

**Figure 4:**
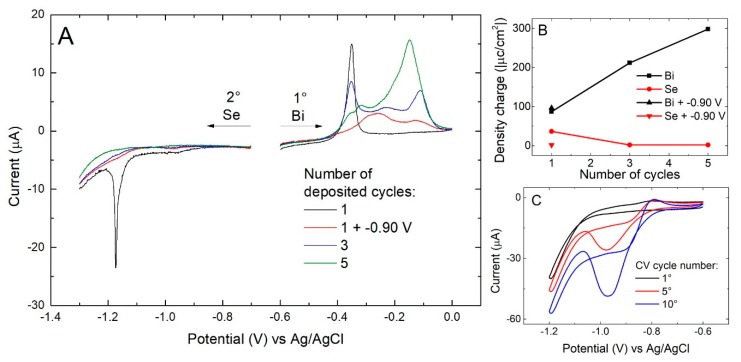
(**A**) Stripping of the Se/Bi_n_, with n equal to the number of the cycles performed (from 1 to 5), first removing the metal and then the non-metal element, 10 mV/s scan rate; (**B**) charge calculated integrating the stripping curves; (**C**) consecutive CVs cycles of selenite on bulk Bi: 1st black, 5th red, 10th blue, 50 mV/s scan rate.

To understand how the deposition process varies as a function of the number of cycles and why we did not obtain the expected quantity of Se in the 20 cycle sample, we performed the stripping of multiple layers (Figure 4A). We found that after the first Se/Bi cycle, not only did the quantity of Se stop growing, but it even decreased by a few fractions of monolayers (Figure 4B). On the other hand, the Bi charge continued to grow, as if the limited presence of Se was still sufficient to induce the SLR of Bi and enable formation of the subsequent layers, otherwise prevented due to the nature of SLR deposition.

This observation suggests a Selective Electrodesorption Based Atomic Layer Deposition (SEBALD)-like [29,30,31] mechanism. SEBALD is a method that allows the deposition of metal clusters or alloys under high morphological control. This technique consists of the E-ALD deposition of multiple layers of one or more metals alternated with an anion. The anion is then reduced and stripped out from the solid, leaving the metal deposit with shapes and/or composition not achievable with a conventional overpotential deposition. In this case, it seems that the SEBALD process occurs even if we want to avoid the chalcogenide electrodesorption. The morphology of this kind of deposit is crystalline, ordered and oriented as we demonstrated, with Atomic Force Microscope (AFM) and Scanning Electron Microscope (SEM) images, in a previous study [31].

The trend of the CV of Se on bulk Bi (Figure 4C) is similar to that one observed on Ag (Figure 1) but the current is much less and a broader peak is observed, suggesting sluggish deposition rate or disorder in the substrate. Assuming that the deposition conditions used for the deposition of the first Se layer affect the subsequent layers, a potential of −0.90 V was applied to the deposit obtained after a single Se/Bi cycle in the presence of an ammonia buffer and then stripping was carried out. Instead of obtaining the typical quantity of Se_ad_, a much lower quantity was observed; it appears, thus, that it is the same deposition potential (−0.90 V) of the first Se layer which, in the presence of Bi, selectively electrodesorbs the deposited Se, probably in the form of selenide.

**Figure 5:**
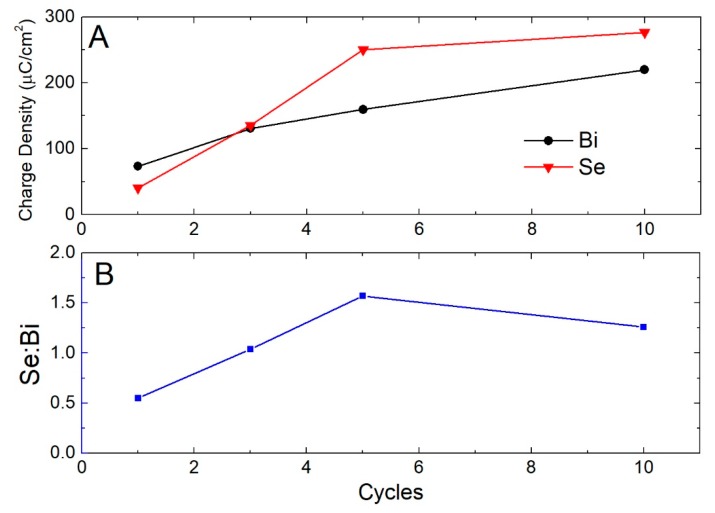
(**A**) Stripped charge, from samples with up to 10 layers, before of the Bi (black) and then of the Se (red); (**B**) ratio between the quantity of Se and the Bi (ideal 1.5).

After performing further experiments, it emerged that, although it was not possible to deposit SLR layers of Se on Bi, the deposition of massive Se remained unaltered and did not interfere with the already deposited underlying Se. This behavior is probably due to the fact that bulk Se is deposited at a lower potential (−0.8 V), without occurring in the reduction and desorption of the underlying layers. In order to deposit an amount of massive Se equivalent to the amount of a Se_ad_ we found that the potential should be kept at −0.8 V for around 3 min (time-controlled deposition). Deposits up to 10 cycles were thus made (Figure 5) and subsequent stripping confirms the presence of both elements. From these measurements we can also obtain a preliminary quantification of the Se:Bi ratio which is close to the stoichiometric value of 3:2. Slight variations are reasonalbly due to the uncontrolled nature of the massive deposition.

**Figure 6:**
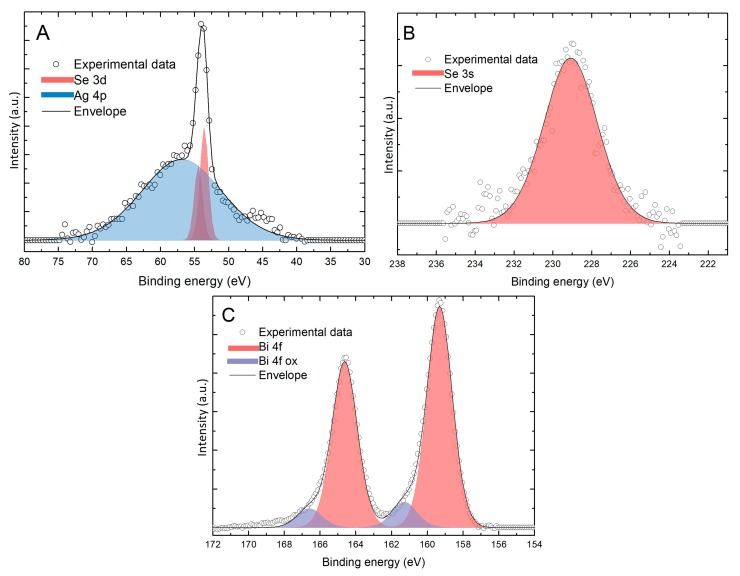
XPS peak of (**A**) 3d transition region of Se and Ag 4p; (**B**) 3s transition region of Se and (**C**) 4f transition region of Bi in the sample prepared performing 20 deposition cycles with the time-controlled bulk Se deposition method.

XPS measurements (Figure 6) were also performed on a sample on which 20 cycles have been grown using the bulk Se deposition method. In Figure 6 the presence of Se is confirmed in both the regions. In particular, the peak relative to the 3d transition is well defined over the Ag 4p transition peak and the position was determined at 53.6 eV, compatible with the expected value for this element in selenide form (52.9 eV, [25,32]). The position of the 4f transition peak of Bi was unchanged and located at the energy expected for Bi_2_O_3_ (159.3 eV [25,32]) and not of Bi_2_Se_3_ (158.1 eV [25,32]). The amount (ratios of the XPS areas) of Bi 4f (42%) and Se 3s (58%) in the sample are compatible with the stoichiometry (2:3) of the desired compound, confirming the electrochemical results (Figure 5). These results tell us that, even if the right amount of bismuth and selenite was deposited on the electrode, the desired compound does not form, instead Ag_2_Se and Bi_2_O_3_ are formed, excluding the bulk deposition as a useful method for the synthesis of this material.

**Figure 7:**
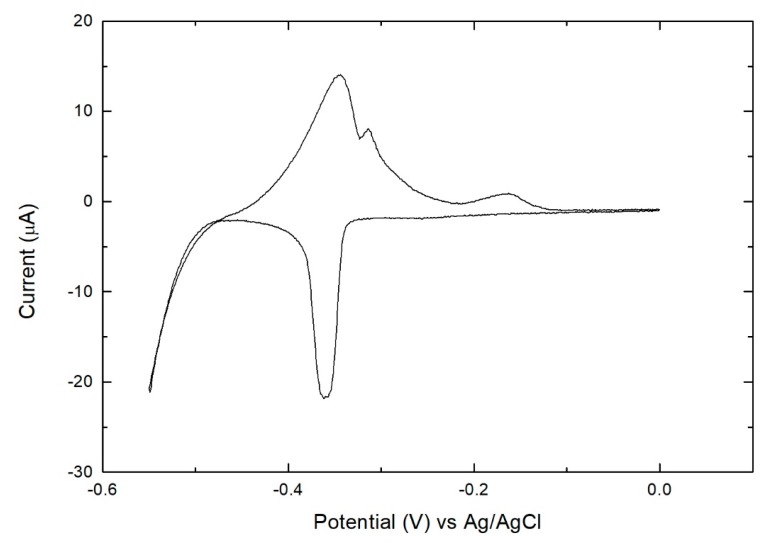
CV of the Bi solution at Ag (111) electrode between 0.0 and −0.55 V at 2 mV/s.

The shape and the position of the Bi stripping peaks in Figure 4A are very different whether in the presence or not of Se (with a shift towards more positive potentials), suggesting a strong Bi interaction with the Ag substrate and the probable formation of alloys. On this basis we evaluate the possibility of depositing a single Bi monolayer directly on the Ag electrode. From the voltammetry of the Bi solution on the Ag electrode (Figure 7), not only does it seem possible to deposit a metal monolayer through SLR, but the deposition peak appears much sharper and more distant from the massive deposition than in the presence of Se. This could be an additional indication for the formation of an Ag/Bi alloy.

**Figure 8:**
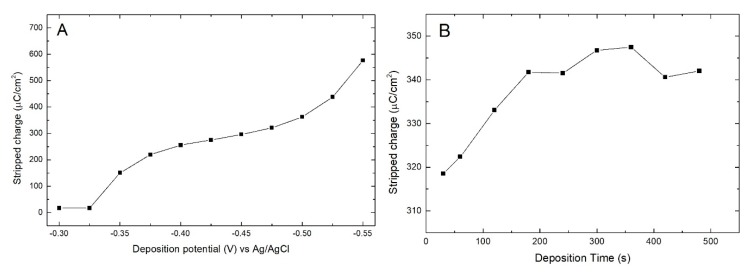
(**A**) Growth of the amount of Bi deposited in one minute on Ag electrode in function of the deposition potential; (**B**) amount of deposited Bi with a potential fixed at −0.45 V depending on the deposition time.

At this point we proceed to the SLR study quantifying the amount of Bi deposited in one minute as a function of the potential (between −0.30 V and −0.55 V) (Figure 8A). The presence of a plateau at intermediate potentials confirms that what is shown in the CV is actually a SLR peak. Holding the potential fixed at −0.45 V, the amount of charge deposited as a function of the deposition time was measured (Figure 8B). The charge tends to grow in the first three minutes, confirming that the monolayer is formed relatively slowly (generally a few tens of seconds are sufficient) and by applying less negative potentials the growth is even slower. Over three minutes of deposition the amount of Bi remains stable, reaching a plateau indicating that bulk Bi is not deposited. Subsequently, Se deposition tests were performed on this Ag/Bi_ad_ layer, but we have no evidence of any SLR.

## 4. Conclusions

In this paper several aspects of the growth of Bi thin films under SLR conditions were investigated. Firstly, the possibility of depositing the metal under controlled conditions on a Se coated silver single crystal was investigated, achieving a single Bi_2_Se_3_ monolayer. Proceeding with the successive layers, it has been observed that the growth of the compound is limited to the first monolayer but the Bi growth is still evident, highlighting the possibility of obtaining multilayers of the metal by means of a SEBALD process. Operating a time-controlled deposition for Se at the bulk potential, we obtained both elements in the final sample, but it is not clear if Bi_2_Se_3_ is formed. Even if the 2:3 stoichiometry ratio between the two elements is confirmed, the observed XPS energy levels do not match with the values available in literature for Bi_2_Se_3_, and for this reason further investigation will be carried out.

Subsequently, the SLR deposition of Bi directly on Ag was also investigated, resulting in a likely formation of an alloy at a potential of −0.45 V. As expected, from the previous measurements, we did not observe any deposition of Se on this type of substrate.

## References

[1] Wood C. (1988). Materials for thermoelectric energy conversion. Inst. Phys..

[2] Zhang H., Liu C.-X., Qi X.-L., Dai X., Fang Z., Zhang S.-C. (2009). Topological insulators in Bi_2_Se_3_, Bi_2_Te_3_ and Sb2Te3 with a single Dirac cone on the surface. Nat. Phys..

[3] Tumelero M.A., Faccio R., Pasa A.A. (2016). Unraveling the native conduction of trichalcogenides and its ideal band alignment for new photovoltaic interfaces. J. Phys. Chem. C.

[4] Tumelero M.A., Benetti L.C., Isoppo E., Faccio R., Zangari G., Pasa A.A. (2016). Electrodeposition and ab Initio Studies of Metastable Orthorhombic Bi_2_Se_3_: A Novel Semiconductor with Bandgap for Photovoltaic Applications. J. Phys. Chem. C.

[5] Ahmed R., Xu Y., Sales M.G., Lin Q., McDonnell S.J., Zangari G. (2018). Synthesis and Material Properties of Bi_2_Se_3_ Nanostructures Deposited by SILAR. J. Phys. Chem. C.

[6] Gregory B.W., Stickney J.L. (1991). Electrochemical atomic layer epitaxy (ECALE). J. Electroanal. Chem. Interfacial Electrochem..

[7] Giaccherini A., Felici R., Innocenti M. (2017). Operando structural characterization of the E-ALD process ultra-thin films growth. X-ray Characterization of Nanostructured Energy Materials by Synchrotron Radiation.

[8] Giaccherini A., Russo F., Carlà F., Guerri A., Picca R.A., Cioffi N., Cinotti S., Montegrossi G., Passaponti M., Di Benedetto F. (2018). Operando SXRD of E-ALD deposited sulphides ultra-thin films: Crystallite strain and size. Appl. Surf. Sci..

[9] Giaccherini A., Cinotti S., Guerri A., Carlà F., Montegrossi G., Vizza F., Lavacchi A., Felici R., Di Benedetto F., Innocenti M. (2017). Operando SXRD study of the structure and growth process of Cu2S ultra-thin films. Sci. Rep..

[10] Giaccherini A., Montegrossi G., Di Benedetto F., Innocenti M. (2018). Thermochemistry of the E-ALD process for the growth of Cu_x_Zn_y_S on Ag(111): Interpretation of experimental data. Electrochim. Acta.

[11] Caporali S., Tolstogouzov A., Teodoro O.M.N.D., Innocenti M., Di Benedetto F., Cinotti S., Picca R.A., Sportelli M.C., Cioffi N. (2015). Sn-deficiency in the electrodeposited ternary Cu_x_Sn_y_S_z_ thin films by ECALE. Sol. Energy Mater. Sol. Cells.

[12] Innocenti M., Becucci L., Bencistà I., Carretti E., Cinotti S., Dei L., Di Benedetto F., Lavacchi A., Marinelli F., Salvietti E. (2013). Electrochemical growth of Cu-Zn sulfides. J. Electroanal. Chem..

[13] Innocenti M., Bencistà I., Bellandi S., Bianchini C., Di Benedetto F., Lavacchi A., Vizza F., Foresti M.L. (2011). Electrochemical layer by layer growth and characterization of copper sulfur thin films on Ag(111). Electrochim. Acta.

[14] Foresti M.L., Milani S., Loglio F., Innocenti M., Pezzatini G., Cattarin S. (2005). Ternary CdS_x_Se_1-x_ deposited on Ag(111) by ECALE: Synthesis and characterization. Langmuir.

[15] Loglio F., Innocenti M., D’Acapito F., Felici R., Pezzatini G., Salvietti E., Foresti M.L. (2005). Cadmium selenide electrodeposited by ECALE: Electrochemical characterization and preliminary results by EXAFS. J. Electroanal. Chem..

[16] Cecconi T., Atrei A., Bardi U., Forni F., Innocenti M., Loglio F., Foresti M.L., Rovida G. (2001). X-ray photoelectron diffraction (XPD) study of the atomic structure of the ultrathin CdS phase deposited on Ag(111) by electrochemical atomic layer epitaxy (ECALE). J. Electron Spectrosc. Relat. Phenom..

[17] Cavallini M., Facchini M., Albonetti C., Biscarini F., Innocenti M., Loglio F., Salvietti E., Pezzatini G., Foresti M.L. (2007). Two-dimensional self-organization of CdS ultra thin films by confined electrochemical atomic layer epitaxy growth. J. Phys. Chem. C.

[18] Xiao C., Yang J., Zhu W., Peng J., Zhang J. (2009). Electrodeposition and characterization of Bi_2_Se_3_ thin films by electrochemical atomic layer epitaxy (ECALE). Electrochim. Acta.

[19] Forni F., Innocenti M., Pezzatini G., Foresti M.L. (2000). Electrochemical aspects of CdTe growth on the face (111) of silver by ECALE. Electrochim. Acta.

[20] Foresti M.L., Capolupo F., Innocenti M., Loglio F. (2002). Visual Detection of Crystallographic Orientations of Face-Centered Cubic Single Crystals. Cryst. Growth Des..

[21] Innocenti M., Cattarin S., Cavallini M., Loglio F., Foresti M.L. (2002). Characterisation of thin films of CdS deposited on Ag(111) by ECALE. A morphological and photoelectrochemical investigation. J. Electroanal. Chem..

[22] Shirley D.A. (1972). High-resolution x-ray photoemission spectrum of the valence bands of gold. Phys. Rev. B.

[23] Susi T., Pichler T., Ayala P. (2015). X-ray photoelectron spectroscopy of graphitic carbon nanomaterials doped with heteroatoms. Beilstein J. Nanotechnol..

[24] Pezzatini G., Loglio F., Innocenti M., Foresti M.L. (2003). Selenium(IV) Electrochemistry on Silver: A Combined Electrochemical Quartz-Crystal Microbalance and Cyclic Voltammetric Investigation. Collect. Czech. Chem. Commun..

[25] NIST NIST X-ray Photoelectron Spectroscopy Database. Srdata.nist.gov/xps.

[26] Dharmadhikari V.S., Sainkar S.R., Badrinarayan S., Goswami A. (1982). Characterisation of thin films of bismuth oxide by X-ray photoelectron spectroscopy. J. Electron Spectrosc. Relat. Phenom..

[27] Innocenti M., Forni F., Pezzatini G., Raiteri R., Loglio F., Foresti M.L. (2001). Electrochemical behavior of As on silver single crystals and experimental conditions for InAs growth by ECALE. J. Electroanal. Chem..

[28] Hatchett D.W., Gao X., Catron S.W., White H.S. (1996). Electrochemistry of sulfur adlayers on Ag(111). Evidence for a concentration- and potential-dependent surface-phase transition. J. Phys. Chem..

[29] Innocenti M., Bellandi S., Lastraioli E., Loglio F., Foresti M.L. (2011). Selective electrodesorption based atomic layer deposition (SEBALD): A novel electrochemical route to deposit metal clusters on Ag(111). Langmuir.

[30] Innocenti M., Zangari G., Zafferoni C., Bencistà I., Becucci L., Lavacchi A. (2013). Selective electrodesorption based atomic layer deposition (SEBALD) modifications of silver surfaces for enhancing oxygen reduction reaction activity. J. Power Sources.

[31] Giurlani W., Giaccherini A., Salvietti E., Passaponti M., Comparini A., Morandi V., Liscio F., Cavallini M., Innocenti M. (2018). Selective Electrodesorption Based Atomic Layer Deposition (SEBALD) of Bismuth under morphological control. Electrochem. Soc. Interface.

[32] Nascimento V.B., de Carvalho V.E., Paniago R., Soares E.A., Ladeira L.O., Pfannes H.D. (1999). XPS and EELS study of the bismuth selenide. J. Electron Spectrosc. Relat. Phenom..

